# Age at Menopause and Development of Type 2 Diabetes in Korea

**DOI:** 10.1001/jamanetworkopen.2024.55388

**Published:** 2025-01-21

**Authors:** Byung-Joon Ko, Jin-Hyung Jung, Kyungdo Han, Ga Eun Nam

**Affiliations:** 1 Joon 365 Clinic, Hwaseong, Republic of Korea; 2 Samsung Biomedical Research Institute, Sungkyunkwan University School of Medicine, Suwon, Republic of Korea; 3 Department of Statistics and Actuarial Science, Soongsil University, Seoul, Republic of Korea; 4 Department of Family Medicine, Korea University Guro Hospital, Korea University College of Medicine, Seoul, Republic of Korea

## Abstract

**Importance:**

There is limited evidence regarding the association between age at menopause and incident type 2 diabetes (T2D).

**Objective:**

To investigate whether age at menopause and premature menopause are associated with T2D incidence in postmenopausal Korean women.

**Design, Setting, and Participants:**

This population-based cohort study was conducted among a nationally representative sample from the Korean National Health Insurance Service database of 1 125 378 postmenopausal women without T2D who enrolled in 2009. The median (IQR) follow-up was 8.4 (8.1-8.7) years. Data were analyzed in March 2024.

**Exposures:**

Age at menopause and premature menopause (menopause onset at age <40 years).

**Main Outcomes and Measures:**

The primary outcome was incident T2D. Multivariable Cox proportional hazards regression analysis was used to estimate hazard ratios (HRs) and 95% CIs for incident T2D by age at menopause, adjusting for potential confounders.

**Results:**

Of 1 125 378 participants (mean [SD] age at enrollment, 61.2 [8.4] years), 113 864 individuals (10.1%) were diagnosed with T2D at least 1 year after enrollment. Women with menopause onset at ages younger than 40 years (premature menopause; HR, 1.13; 95% CI, 1.08-1.18) and ages 40 to 44 years (HR, 1.03; 95% CI, 1.00-1.06) had increased risk of T2D compared with those with onset at age 50 years or older, with adjustment for sociodemographic, lifestyle, cardiometabolic, psychiatric, and reproductive factors; a younger age at menopause was associated with increased risk of developing T2D (*P* for trend <.001). Body mass index, depressive disorder, and prediabetes modified the association in subgroup analyses; for example, for individuals with premature menopause vs those with menopause at ages 50 years or older, HRs were 1.54 (95% CI, 1.14-2.06) for a BMI less than 18.5 and 1.14 (95% CI, 1.00-1.30) for a BMI of 30 or greater (*P* < .001), 1.28 (95% CI, 1.12-1.45) for individuals with depression and 1.11 (95% CI, 1.07-1.16) for those without depression (*P* = .01), and 1.25 (95% CI, 1.18-1.33) for individuals who were not prediabetic and 1.04 (95% CI, 0.99-1.11) those who were prediabetic (*P* < .001).

**Conclusions and Relevance:**

In this study, premature and early menopause were associated with a higher risk of T2D, highlighting the need for targeted public health strategies aimed at preventing or delaying T2D among postmenopausal women.

## Key Points

**Question:**

Are age at menopause or premature menopause associated with risk of type 2 diabetes (T2D)?

**Findings:**

In this cohort study of 1 125 378 postmenopausal women in South Korea, women experiencing premature menopause had a higher risk of T2D compared with those with menopause onset at age 50 years or older. A younger age at menopause was associated with an increased risk of developing T2D.

**Meaning:**

This study found that premature or early menopause were associated with a higher risk for the development of T2D, suggesting that prevention targeting women with early menopause may be warranted.

## Teaser

This cohort study investigates whether age at menopause is associated with a subsequent diagnosis of type 2 diabetes among women in Korea.

## Introduction

Type 2 diabetes (T2D) is a chronic disease that leads to cardiovascular diseases, microvascular complications, and increased mortality.^1^ Despite advancements in therapeutic drugs and efforts to diagnose and treat T2D, its prevalence continues to increase, and control rates remain low.^2^ Recently, there has been a greater emphasis on the prevention of T2D, early diagnosis, and stringent glucose control in early stages to prevent or delay complications.^3^ Thus, it is crucial to identify individuals with risk factors associated with T2D and prioritize these individuals for screening over other groups.

Although the prevalence of T2D in young adults is higher in men, it increases in women from menopause to older age.^2^ Genetics, obesity, and lifestyle factors are well-known risk factors associated with T2D; however, research on women-specific risk factors is scarce.^4^

Several studies have shown an association between female reproductive factors and cardiovascular diseases.^5,6^ Specifically, research has explored associations of age at menopause and premature menopause with cardiovascular diseases.^7,8^ Premature menopause, caused by ovarian dysfunction, results in the early loss of protective effects of female hormones, leading to adverse health outcomes, such as cardiovascular diseases, dementia, fractures, and increased mortality.^7,8,9,10^

Previous studies have investigated the association of female reproductive factors, such as breastfeeding and parity, with incident T2D.^11,12^ Specifically, a few studies^13,14,15^ have examined the association of age at menopause and premature menopause with incident T2D. However, these studies were relatively small, used cross-sectional designs, and included limited representation of the Asian population. Consequently, this study aimed to investigate whether age at menopause and premature menopause were associated with incident T2D in more than 1 million Korean women using a longitudinal follow-up study design.

## Methods

This cohort study was approved by the Institutional Review Board of the Korea University Anam Hospital. The board granted an exemption from informed consent given that all data collected for analysis were anonymized and deidentified. The Strengthening the Reporting of Observational Studies in Epidemiology (STROBE) reporting guideline was followed in the course of this study.

### Data Source and Study Population

Data from the Korean National Health Insurance Service (NHIS) were used for this analysis. The NHIS is a mandatory, comprehensive health insurance system covering 97% of the South Korean population. It provides periodic health examinations, including anthropometric measurements, self-administered questionnaires about lifestyle, and laboratory assays, at least every 2 years to nearly all South Koreans. NHIS data encompass demographic information, health checkup records, and diagnoses and treatments coded according to the *International Statistical Classification of Diseases and Related Health Problems, Tenth Revision *(*ICD-10*).

**Figure 1.**
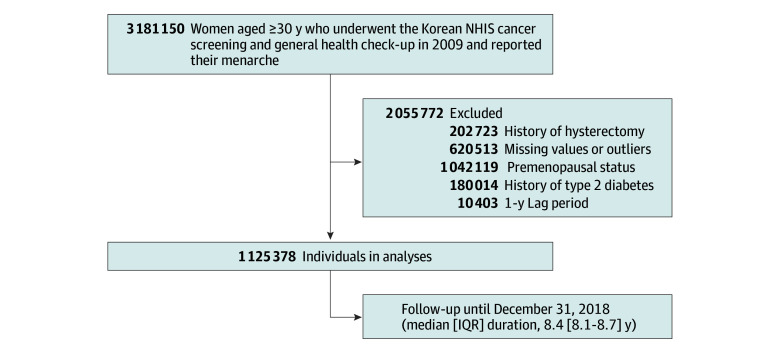
Study Flowchart After excluding participants meeting exclusion criteria, 1 125 378 postmenopausal women were included in the final analysis. NHIS indicates National Health Insurance Service.

A total of 3 181 150 women aged 30 years and older who underwent NHIS cancer screening and general health examinations in 2009 and reported age at menarche were initially included. We excluded 202 723 individuals who had undergone a hysterectomy, 620 513 individuals with outlier ages at menarche (<5 or ≥30 years) or menopause (<30 or >60 years) or missing data on key variables, 1 042 119 individuals who were premenopausal, and 180 014 individuals with a history of T2D or fasting glucose levels of 126 mg/dL or greater (to convert glucose to millimoles per liter, multiply by 0.0555). After a 1-year lag, an additional 10 403 women who developed T2D were excluded to reduce bias associated with undiagnosed T2D at baseline. Ultimately, 1 125 378 women were enrolled and followed up until December 31, 2018 (Figure 1).

### Study End Point

The end point was new-onset T2D from the index date to the end of this study; new-onset T2D was defined as a fasting blood glucose level of 126 mg/dL or greater during an NHIS health examination or a history of claims for antidiabetic medication under *ICD-10* codes E11 to E14. This definition was widely used in previous claims-based studies.^16,17^ Participants were followed up until the occurrence of T2D, death, or December 31, 2018. Given that the NHIS is a mandatory service, the only alternative reason for loss to follow-up was migrating to another country, which was not considered in this study. However, considering the small number of emigrants in Korea, this factor was unlikely to have a significant influence on results.^18^ The median (IQR) follow-up duration was 8.4 (8.1-8.7) years.

### Assessment of Age at Menopause and Premature Menopause

Age at menopause was assessed using a self-administered questionnaire and categorized into 4 groups: younger than 40 years, ages 40 to 44 years, ages 45 to 49 years, and age 50 years and older. Premature menopause was defined as menopause occurring before age 40 years, excluding individuals with a history of hysterectomy.^7,19^

### Covariates

Participant reproductive and lifestyle factors were ascertained using a self-reported questionnaire. Reproductive factors included age at menopause and menarche, parity, breastfeeding, oral contraceptive use, and menopausal hormone therapy. Smoking status (current smoker or not), alcohol (alcohol drinker or not), and physical activity (defined as engaging in moderate-intensity exercise ≥5 d/wk or vigorous exercise ≥3 d/wk) were assessed and categorized. Additionally, a low income level was defined as belonging to the lowest income quartile or receiving medical aid based on monthly health insurance premiums. The place of residence was classified as urban or rural.

Anthropometric variables, such as height, weight, waist circumference, and blood pressure, and laboratory parameters, including serum glucose, lipid profile, and creatinine levels, were measured after an overnight fast. Waist circumference was measured at the midline between the lower border of the rib cage and the upper border of the iliac crest. Body mass index (BMI; calculated as weight in kilograms divided by height in meters squared) was assessed, with obesity defined as a BMI of 25 or greater.^20^ BMI was further classified into 5 categories: less than 18.5, 18.5 to 22.9, 23.0 to 24.9, 25.0 to 29.9, and 30.0 or greater.

Hypertension was determined by a systolic over diastolic pressure of 140/90 mm Hg or greater or a claim for antihypertensive medication under *ICD-10* codes I10 to I13 and I15. Dyslipidemia was defined as having a serum total cholesterol of 240 mg/dL or greater (to convert cholesterol to millimoles per liter, multiply by 0.0259) or the presence of lipid-lowering medication claims under *ICD-10* code E78. Chronic kidney disease was identified with an estimated glomerular filtration rate less than 60 mL/min/1.73 m^2^ according to the Modification of Diet in Renal Disease method equation.^21^ Depressive and anxiety disorders were defined using *ICD-10* codes F32, F33, F40, and F41. Prediabetes was defined as a fasting glucose level of 100 to 125 mg/dL.^22^

### Statistical Analysis

Baseline characteristics of the study population are presented as means and SDs for continuous data and as numbers with percentages for categorical data, categorized by age at menopause. Continuous data were analyzed using the 1-way analysis of variance, while categorical data were assessed with the χ^2^ test. Kaplan-Meier curves were used to present cumulative incidence probabilities of T2D based on premature menopause and age at menopause, and these were compared using the log-rank test. The incidence rate of T2D was calculated by dividing the number of events by 1000 person-years. Hazard ratios (HRs) and 95% CIs for the association of premature menopause and age at menopause with T2D were determined using multivariable Cox proportional hazards regression analysis. Model 1 was unadjusted; model 2 was adjusted for age, income, place of residence, smoking status, alcohol consumption, physical activity, BMI category, hypertension, dyslipidemia, chronic kidney disease, depressive disorder, and anxiety disorder. Model 3 included all variables from model 2 plus age at menarche, parity, breastfeeding, oral contraceptive use, and menopausal hormone therapy. *P* values for linear trends according to age at menopause were evaluated. Subgroup analyses stratified by sociodemographic, lifestyle, cardiometabolic, psychiatric, and reproductive variables were conducted to explore interactions between these subgroups and the risk of T2D across menopausal age groups. This was done because previous research has indicated that these variables may interact with the association between menopause age and T2D.^14,15^ All statistical analyses were performed using SAS statistical software version 9.4 (SAS Institute), with a 2-sided *P* < .05 considered statistically significant. Data analysis was conducted in March 2024.

## Results

### Baseline Characteristics

Of 1 125 378 participants (mean age, 61.2 [8.4] years at enrollment and 50.0 [3.9] years at menopause), 113 864 individuals (10.1%) were diagnosed with T2D during the median (IQR) follow-up of 8.4 (8.1-8.7) years. Age at menopause was younger than 40 years (premature menopause) among 19 311 women, ages 40 to 44 years among 64 700 women, ages 45 to 49 years among 310 772 women, and ages 50 or older among 730 595 women. Baseline characteristics across menopausal age categories are presented in Table 1. Women with premature menopause were more likely than women without premature menopause (eg, women with menopause onset at ages ≥50 years) to live in rural areas (urban residence: 6193 individuals [32.1%] vs 324 245 individuals [44.4%]), smoke (790 individuals [4.1%] vs 16 412 individuals [2.3%]), and exercise less (regular exercisers: 2985 individuals [15.5%] vs 138 638 individuals [19.0%]) and had higher rates of obesity (7055 individuals [36.5%] vs 263 085 individuals [36.0%]), hypertension (8155 individuals [42.2%] vs 289 896 individuals [39.7%]), chronic kidney disease (2638 individuals [13.7%]) vs 76 199 individuals [10.4%]), depressive disorder (1432 individuals [7.4%] vs 47 299 individuals [6.5%]), and anxiety disorder (3047 individuals [15.8%]) vs 97 520 individuals [13.4%]) (all *P* < .001). They also had lower rates of parity, breastfeeding, and oral contraceptive use and were more likely to use menopausal hormone therapy than women without premature menopause. Maximum absolute standardized mean differences are shown in eTable 1 in Supplement 1.

**Table 1.** Baseline Characteristics of Study Population

Characteristic	Participants, No. (%)					*P* value
	Total (N = 1 125 378)	Age at menopause, y				
		<40 (n = 19 311)	40-44 (n = 64 700)	45-49 (n = 310 772)	≥50 (n = 730 595)	
Age, mean (SD), y	61.2 (8.4)	63.1 (10.6)	62.4 (10.7)	60.3 (9.2)	61.4 (7.7)	<.001
Income (lowest quartile or medical aid)	243 315 (21.6)	3978 (20.6)	13 490 (20.9)	66 886 (21.5)	158 961 (21.8)	<.001
Urban place of residence	481 212 (42.8)	6193 (32.1)	23 133 (35.8)	127 641 (41.1)	324 245 (44.4)	<.001
Current smoker	28 841 (2.6)	790 (4.1)	2321 (3.6)	9318 (3.0)	16 412 (2.3)	<.001
Alcohol drinker	143 531 (12.8)	2392 (12.4)	8320 (12.9)	42 106 (13.6)	90 713 (12.4)	<.001
Regular exerciser	207 017 (18.4)	2985 (15.5)	10 187 (15.7)	55 207 (17.8)	138 638 (19.0)	<.001
Obesity	396 364 (35.2)	7055 (36.5)	22 615 (35.0)	103 609 (33.3)	263 085 (36.0)	<.001
Hypertension	437 551 (38.9)	8155 (42.2)	26 153 (40.4)	113 347 (36.5)	289 896 (39.7)	<.001
Dyslipidemia	333 892 (29.7)	5596 (29.0)	17 753 (27.4)	87 664 (28.2)	222 879 (30.5)	<.001
Chronic kidney disease	121 483 (10.8)	2638 (13.7)	8580 (13.3)	34 066 (11.0)	76 199 (10.4)	<.001
Depressive disorder	74 303 (6.6)	1432 (7.4)	4756 (7.4)	20 816 (6.7)	47 299 (6.5)	<.001
Anxiety disorder	151 897 (13.5)	3047 (15.8)	9597 (14.8)	41 733 (13.4)	97 520 (13.4)	<.001
Prediabetes	304 749 (27.1)	5148 (26.7)	17 635 (27.3)	82 890 (26.7)	199 076 (27.3)	<.001
BMI, mean (SD)	24.0 (3.1)	24.0 (3.3)	23.9 (3.3)	23.9 (3.1)	24.1 (3.0)	<.001
Waist circumference, mean (SD), cm	79.5 (8.1)	80.1 (8.6)	79.6 (8.5)	79.0 (8.2)	79.6 (8.0)	<.001
Fasting glucose, mean (SD), mg/dL	93.8 (11.1)	93.5 (11.3)	93.8 (11.3	93.6 (11.0)	93.9 (11.1)	<.001
Systolic blood pressure, mean (SD), mm Hg	125.0 (16.0)	125.8 (16.7)	125.4 (16.7)	124.3 (16.1)	125.3 (15.9)	<.001
Diastolic blood pressure, mean (SD), mm Hg	76.8 (10.1)	77.2 (10.4)	76.9 (10.4)	76.5 (10.2)	76.9 (10.1)	<.001
Total cholesterol, mean (SD), mg/dL	208.7 (37.9)	206.8 (38.6)	206.3 (38.3)	207.7 (37.9)	209.3 (37.9)	<.001
Reproductive factors						
Age at menopause, mean (SD), y	50.0 (3.9)	36.8 (2.6)	41.8 (1.5)	47.5 (1.4)	52.1 (2.2)	<.001
Age at menarche, mean (SD), y	16.4 (1.9)	16.9 (2.1)	16.6 (2.0)	16.4 (1.9)	16.4 (1.8)	<.001
Parity	1 105 857 (98.3)	18 784 (97.3)	63 205 (97.7)	304 574 (98.0)	719 294 (98.5)	<.001
Breastfeeding	1 050 768 (93.4)	17 735 (91.8)	59 443 (91.9)	286 634 (92.2)	686 956 (94.0)	<.001
Oral contraceptive use	172 166 (15.3)	2643 (13.7)	9525 (14.7)	48 128 (15.5)	111 870 (15.3)	<.001
Menopausal hormone therapy	187 194 (16.6)	3660 (19.0)	12 181 (18.8)	58 918 (19.0)	112 435 (15.4)	<.001

Abbreviation: BMI, body mass index (calculated as weight in kilograms divided by height in meters squared).

SI conversion factors: To convert cholesterol to millimoles per liter, multiply by 0.0259; glucose to millimoles per liter, multiply by 0.0555.

### Longitudinal Associations of Premature Menopause and Age at Menopause With Incident T2D

**Figure 2.**
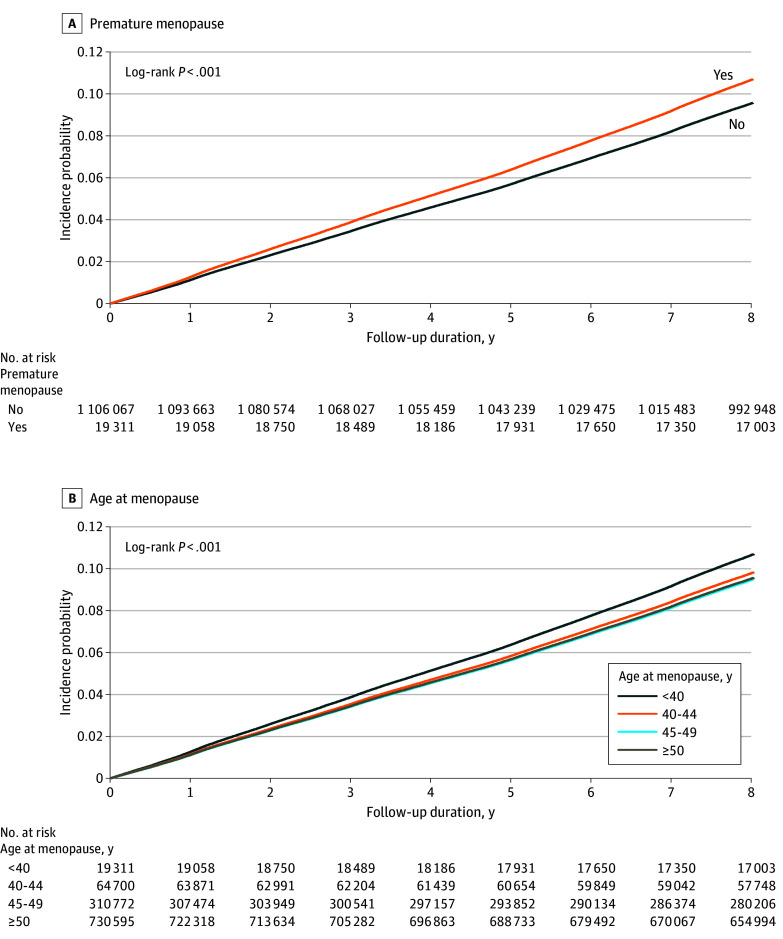
Kaplan-Meier Curves for Incidence Probabilities of Type 2 Diabetes Curves show incidence by premature menopause status (A) and age at menopause (B) and were plotted after adjusting for age, income, place of residence, smoking status, alcohol consumption, physical activity, body mass index category, hypertension, dyslipidemia, chronic kidney disease, depressive disorder, anxiety disorder, age at menarche, parity, breastfeeding, oral contraceptive use, and menopausal hormone therapy.

During the median (IQR) follow-up of 8.4 (8.1-8.7) years, 113 864 individuals (10.1%) developed T2D. Of these, 2337 individuals (12.1%) were in the premature menopause group and there were 6912 individuals (10.7%), 30 008 individuals (9.7%), and 74 607 individuals (10.2%) in groups aged 40 to 44 years, 45 to 49 years, and 50 years or older at menopause, respectively (Table 2). The cumulative incidence of T2D was higher in women with vs those without premature menopause (log-rank *P* < .001) (Figure 2A). As age at menopause decreased, the incidence probability of T2D increased (log-rank *P* < .001) (Figure 2B).

**Table 2.** Association of Menopause Timing With Type 2 Diabetes

Menopause timing	Participants, No.	Events, No.	Person-years	IR^a^	Model 1, HR (95% CI)^b^	*P* value	Model 2, HR (95% CI)^b^	*P* value	Model 3, HR (95% CI)^b^	*P* value
Premature menopause										
No	1 106 067	111 527	8 888 369	12.6	1 [Reference]	<.001	1 [Reference]	<.001	1 [Reference]	<.001
Yes	19 311	2337	154 304	15.2	1.21 (1.16-1.26)		1.13 (1.08-1.18)		1.13 (1.08-1.18)	
Age at menopause, y										
<40	19 311	2337	154 304	15.2	1.19 (1.14-1.24)	.09^c^	1.13 (1.08-1.18)	<.001^c^	1.13 (1.08-1.18)	<.001^c^
40-44	64 700	6912	519 820	13.3	1.05 (1.02-1.07)		1.03 (1.00-1.06)		1.03 (1.00-1.06)	
45-49	310 772	30 008	2 504 310	12.0	0.94 (0.93-0.95)		0.99 (0.98-1.01)		0.99 (0.98-1.01)	
≥50	730 595	74 607	5 864 239	12.7	1 [Reference]		1 [Reference]		1 [Reference]	

Abbreviations: HR, hazard ratio; IR, incidence rate.

^a^ Incidence per 1000 person-years.

^b^ Model 1 was not adjusted. Model 2 was adjusted for age, income, place of residence, smoking status, alcohol consumption, physical activity, body mass index category, hypertension, dyslipidemia, chronic kidney disease, depressive disorder, and anxiety disorder. Model 3 was adjusted for the same factors as model 2 plus age at menarche, parity, breastfeeding, oral contraceptive use, and menopausal hormone therapy. HRs (95% CIs) were calculated using multivariable Cox proportional hazards regression analysis.

^c^ *P* for trend.

The association between age at menopause and T2D risk is detailed in Table 2. Women who experienced premature menopause had an increased risk of T2D (HR, 1.13; 95% CI, 1.08-1.18) compared with those without premature menopause after adjusting for all confounding factors (model 3). Compared with the group with menopause at ages 50 years older, HRs for groups aged 45 to 49, 40-44, and <40 years were 0.99 (95% CI, 0.98-1.01), which was not significant; 1.03 (95% CI, 1.00-1.06); and 1.13 (95% CI, 1.08-1.18), respectively. A younger age at menopause was associated with an increased risk of T2D (*P* for trend <.001). We additionally analyzed the risk of T2D by 3 categories of age at menopause (<40, 40-44, ≥45 years), and the inverse association between age at menopause and T2D risk was consistently observed (eTable 2 in Supplement 1).

### Subgroup Analysis

Subgroup analyses on the association between age at menopause and incident T2D by sociodemographic, lifestyle, cardiometabolic, psychiatric, and reproductive factor are presented in Table 3. Modifiers of the association between age at menopause and T2D risk included BMI categories, depressive disorder, and prediabetes. Specifically, HRs in this association were higher in groups without obesity, with depressive disorder, and who were not prediabetic. For example, for individuals with premature menopause vs those with menopause at ages 50 years or older, HRs were 1.54 (95% CI, 1.14-2.06) for a BMI less than 18.5 and 1.14 (95% CI, 1.00-1.30) for a BMI of 30 or greater (*P* < .001), 1.28 (95% CI, 1.12-1.45) for individuals with depression and 1.11 (95% CI, 1.07-1.16) for those without depression (*P* = .01), and 1.25 (95% CI, 1.18-1.33) for individuals who were not prediabetic and 1.04 (95% CI, 0.99-1.11) for those who were prediabetic (*P* < .001). No interactions were observed for age, income, place of residence, lifestyle factors, hypertension, dyslipidemia, chronic kidney disease, parity, breastfeeding, oral contraceptive use, or history of menopausal hormone therapy.

**Table 3.** Association of Age at Menopause With Type 2 Diabetes by Patient Characteristic

Characteristic	HR (95% CI)^a^				*P* for interaction
	<40 y^b^	40-44 y^b^	45-49 y^b^	≥50 y^b^	
Age, y					
30-64	1.18 (1.11-1.26)	1.04 (1.00-1.08)	1.00 (0.98-1.02)	1 [Reference]	.11
≥65	1.08 (1.02-1.14)	1.01 (0.98-1.05)	0.98 (0.96-1.00)	1 [Reference]	
Income					
Quartile 1 or medical aid	1.18 (1.08-1.29)	1.04 (0.99-1.10)	0.99 (0.96-1.02)	1 [Reference]	.66
Quartile 2-4	1.11 (1.06-1.17)	1.03 (1.00-1.05)	0.99 (0.98-1.01)	1 [Reference]	
Place of residence					
Urban	1.13 (1.08 1.19)	1.02 (0.99 1.06)	1.00 (0.98 1.02)	1 [Reference]	.56
Rural	1.12 (1.04 1.20)	1.04 (1.00 1.08)	0.98 (0.96 1.00)	1 [Reference]	
Smoking status					
Nonsmoker	1.13 (1.08-1.18)	1.03 (1.01-1.06)	0.99 (0.98-1.01)	1 [Reference]	.47
Current smoker	1.08 (0.90-1.30)	0.95 (0.84-1.07)	1.00 (0.94-1.08)	1 [Reference]	
Alcohol consumption					
No	1.12 (1.07-1.17)	1.03 (1.01-1.06)	0.99 (0.98-1.01)	1 [Reference]	.55
Yes	1.21 (1.07-1.37)	1.00 (0.93-1.08)	0.99 (0.95-1.03)	1 [Reference]	
Regular physical activity					
No	1.13 (1.08-1.18)	1.03 (1.01-1.06)	0.99 (0.98-1.01)	1 [Reference]	.81
Yes	1.11 (1.00-1.24)	1.01 (0.95-1.08)	1.00 (0.97-1.03)	1 [Reference]	
BMI					
<18.5	1.54 (1.14-2.06)	1.11 (0.92-1.35)	1.04 (0.92-1.18)	1 [Reference]	<.001
18.5-22.9	1.32 (1.21-1.43)	1.05 (1.00-1.11)	0.96 (0.93-0.98)	1 [Reference]	
23-24.9	1.05 (0.96-1.15)	1.03 (0.98-1.09)	1.01 (0.98-1.04)	1 [Reference]	
25-29.9	1.06 (1.00-1.13)	1.01 (0.97-1.04)	1.00 (0.98-1.02)	1 [Reference]	
≥30	1.14 (1.00-1.30)	1.06 (0.98-1.15)	1.00 (0.95-1.05)	1 [Reference]	
Hypertension					
No	1.17 (1.10-1.25)	1.04 (1.00-1.08)	1.01 (0.99-1.03)	1 [Reference]	.13
Yes	1.09 (1.04-1.16)	1.02 (0.99-1.05)	0.98 (0.96-1.00)	1 [Reference]	
Dyslipidemia					
No	1.11 (1.05-1.17)	1.04 (1.01-1.08)	0.99 (0.97-1.01)	1 [Reference]	.36
Yes	1.15 (1.08-1.23)	1.01 (0.97-1.05)	1.00 (0.98-1.02)	1 [Reference]	
Chronic kidney disease					
No	1.13 (1.08-1.18)	1.03 (1.00-1.06)	1.00 (0.98-1.01)	1 [Reference]	.22
Yes	1.13 (1.02-1.25)	1.02 (0.95-1.08)	0.96 (0.92-0.99)	1 [Reference]	
Depressive disorder					
No	1.11 (1.07-1.16)	1.02 (1.00-1.05)	0.99 (0.97-1.00)	1 [Reference]	.01
Yes	1.28 (1.12-1.45)	1.08 (0.99-1.17)	1.06 (1.01-1.10)	1 [Reference]	
Prediabetes					
No	1.25 (1.18-1.33)	1.10 (1.07-1.15)	1.01 (0.99-1.04)	1 [Reference]	<.001
Yes	1.04 (0.99-1.11)	0.96 (0.93-0.99)	0.97 (0.95-0.98)	1 [Reference]	
Parity					
No	1.38 (1.04-1.83)	1.03 (0.85-1.25)	0.97 (0.87-1.09)	1 [Reference]	.50
Yes	1.12 (1.08-1.17)	1.03 (1.00-1.06)	0.99 (0.98-1.01)	1 [Reference]	
Breastfeeding					
No	1.22 (1.04-1.43)	0.97 (0.88-1.07)	0.94 (0.89-0.99)	1 [Reference]	.08
Yes	1.12 (1.07-1.17)	1.03 (1.01-1.06)	1.00 (0.98-1.01)	1 [Reference]	
Use of oral contraceptives					
No	1.13 (1.09-1.19)	1.04 (1.01-1.07)	1.00 (0.98-1.01)	1 [Reference]	.38
Yes	1.09 (0.97-1.21)	0.98 (0.92-1.05)	0.98 (0.95-1.01)	1 [Reference]	
Menopausal hormone therapy					
No	1.12 (1.07-1.17)	1.04 (1.01-1.06)	1.00 (0.98-1.01)	1 [Reference]	.41
Yes	1.16 (1.04-1.28)	0.99 (0.93-1.06)	0.97 (0.94-1.01)	1 [Reference]	

Abbreviations: BMI, body mass index (calculated as weight in kilograms divided by height in meters squared); HR, hazard ratio.

^a^ HRs (95% CIs) were calculated using multivariable Cox proportional hazards regression analysis after adjusting for age, income, place of residence, smoking status, alcohol consumption, physical activity, body mass index categories, hypertension, dyslipidemia, chronic kidney disease, depressive disorder, anxiety disorder, age at menarche, parity, breastfeeding, oral contraceptive use, and menopausal hormone therapy.

^b^ Age categories are age at menopause.

## Discussion

In this large-scale cohort study of more than 1.1 million Korean women, premature menopause was associated with an increased risk of T2D after adjusting for conventional cardiovascular risk factors. A younger age at menopause was associated with an increased risk of developing T2D.

Early menopause or premature ovarian insufficiency leads to a longer period without estrogen compared with normal or late menopause. Estrogens reduce oxidative stress and inflammation in adipose tissue and protect against insulin resistance in female mice.^23^ Furthermore, estrogen plays a crucial role in long-term insulin secretion and glucose homeostasis.^24^ Therefore, a deficiency in estrogen may be associated with the risk of T2D in clinical scenarios, such as early menopause. Moreover, early menopause may prompt premature aging, which is associated with DNA damage and contributes to metabolic dysfunction, including T2D.^25^ Previous studies showed that an early onset of natural menopause was associated with a higher risk of T2D, corroborating the findings of this study.^14,15^ Other research found that early and late menopause were associated with an increased T2D risk, which the authors attributed to adverse glucose metabolism outcomes due to short- and long-term estrogen exposure.^26,27^ Studies in the US, Europe, and China suggested no interaction of race and ethnicity in the association between age at menopause and T2D risk.^14,15,26,27^ However, a 2023 pooled analysis^28^ indicated that this association may be less pronounced in South or Southeast Asian, Black, and multiracial or multiethnic groups.

We also found that BMI, depressive disorder, and prediabetes modified the association between age at menopause and T2D risk. The increase in T2D risk associated with early menopause was more pronounced in individuals without obesity, those with depressive disorder, and nonprediabetic groups. Postmenopausal women with obesity have higher serum estradiol concentrations than those without obesity,^29^ suggesting that the increase in T2D risk associated with early menopause was lower in the group with obesity, likely due to the protective effect of estrogen. Depressive disorder may be associated with increased T2D risk by promotion of unhealthy behaviors, such as consuming a high-calorie diet and engaging in less physical activity.^30^ Prediabetes progresses to T2D at a rate 4.7 to 12.1 times higher than the occurrence of T2D in the normoglycemic group,^31^ indicating that the association of early menopause with T2D may be more noticeable in the nonprediabetic group than the prediabetic group.

Given that premature menopause is already considered a risk factor associated with cardiovascular disease in the recent cholesterol guidelines from the American College of Cardiology and American Heart Association,^32^ we suggest that it should also be recognized as a risk factor associated with T2D in diabetes management guidelines. The history of premature menopause could serve as a factor associated with enhanced risk for T2D and could be used to screen for at-risk groups given that the association between early menopause and incidence of T2D has been consistently verified in several prospective studies^14,15,27^ and a meta-analysis.^33^ Early diagnosis and timely management of T2D are crucial to prevent complications associated with the disease.^3^

### Limitations and Strengths

This study has several limitations. First, we collected data on menopause using a self-reported questionnaire rather than medical records or direct observations; therefore, recall bias may have occurred. However, the memory of age at menopause is relatively accurate,^34^ and considering that the outcome was prospectively assessed, this type of exposure could lead to nondifferential misclassification, which tends to bias the effect of exposure toward the null. Second, T2D was ascertained by a fasting serum glucose level of 126 mg/dL or greater or by the presence of a T2D *ICD-10* code diagnosed by a doctor. Glycosylated hemoglobin levels were not used to diagnose T2D; therefore, there may be a misclassification of populations with undiagnosed T2D. Moreover, the Korean NHIS operates on a fee-for-service insurance reimbursement system, which may encourage doctors to exaggerate diagnoses to receive reimbursement, possibly leading to an overestimation of the outcome. However, such errors are random and tend to attenuate observed associations. Third, only participants who responded to the questionnaire on female reproductive history were included in this analysis, and this may have introduced a selection bias. Fourth, it was challenging to determine the varying associations of mental health disorders due to difficulties in classifying them into major depressive disorder, depressive episodes, and specific types of anxiety disorders.

Despite these limitations, the large sample size of 1.1 million postmenopausal women enabled us to consider various confounding variables and effect modifications based on sociodemographic, lifestyle, cardiometabolic, psychiatric, and reproductive factors. Furthermore, based on a well-established and validated longitudinal national database, our findings underscore the importance of women-specific factors, such as age at menopause and premature menopause, which could be associated with the risk of T2D beyond conventional risk factors.

## Conclusions

This cohort study found that premature menopause was associated with an increased incidence of T2D and that an earlier age at menopause was associated with increased risk of developing T2D in a dose-dependent manner. These findings suggest that premature menopause should be emphasized and considered an emerging risk factor in the management of T2D to delay disease progression and inform therapeutic strategies.

## Notes

Supplement 1. **eTable 1.** Baseline characteristics of study population (including MASD) **eTable 2.** Hazard ratios (95%CIs) of type 2 diabetes according to age at menopause (3 categories)

Supplement 2.
Data Sharing Statement
