# Supplementary material for: Age at Menopause and Development of Type 2 Diabetes in Korea
Source: JAMA Netw Open. 2025 Jan 21;8(1):e2455388. doi: 10.1001/jamanetworkopen.2024.55388 (PMC11751743; doi:10.1001/jamanetworkopen.2024.55388)
Supplement: Supplement 1. — eTable 1. Baseline characteristics of study population (including MASD) eTable 2. Hazard ratios (95%CIs) of type 2 diabetes according to age at menopause (3 categories) [file jamanetwopen-e2455388-s001.pdf]

Supplemental Online Content

Ko B-J, Jung J-H, Han K, Nam GE. Age at menopause and development of type 2 diabetes in Korea. *JAMA Netw Open*. 2025;8(1):e2455388.
doi:10.1001/jamanetworkopen.2024.55388

eTable 1. Baseline characteristics of study population (including MASD)

eTable 2. Hazard ratios (95%CIs) of type 2 diabetes according to age at menopause (3 categories)

This supplemental material has been provided by the authors to give readers additional information about their work.

© 2024 Ko B-J et al. *JAMA Network Open*.

eTable 1. Baseline characteristics of study population (including MASD)

	Total	Age at menopause (years)				P	MASD
		<40	40–44	45−49	≥50		
N	1,125,378	19,311	64,700	310,772	730,595		
Age (years)	61.2 ± 8.4	63.1 ± 10.6	62.4 ± 10.7	60.3 ± 9.2	61.4 ± 7.7	<.001	.29
Income (lowest quartile or Medical Aid)	243,315 (21.6)	3978 (20.6)	13,490 (20.9)	66,886 (21.5)	158,961 (21.8)	<.001	.03
Place of residence (urban)	481,212 (42.8)	6193 (32.1)	23,133 (35.8)	127,641 (41.1)	324,245 (44.4)	<.001	.26
Current smokers	28,841 (2.6)	790 (4.1)	2321 (3.6)	9318 (3.0)	16,412 (2.3)	<.001	.11
Alcohol drinkers	143,531 (12.8)	2392 (12.4)	8320 (12.9)	42,106 (13.6)	90,713 (12.4)	<.001	.04
Regular exercisers	207,017 (18.4)	2985 (15.5)	10,187 (15.7)	55,207 (17.8)	138,638 (19.0)	<.001	.09
Obesity	396,364 (35.2)	7055 (36.5)	22,615 (35.0)	103,609 (33.3)	263,085 (36.0)	<.001	.07
Hypertension	437,551 (38.9)	8155 (42.2)	26,153 (40.4)	113,347 (36.5)	289,896 (39.7)	<.001	.12
Dyslipidemia	333,892 (29.7)	5596 (29.0)	17,753 (27.4)	87,664 (28.2)	222,879 (30.5)	<.001	.07
Chronic kidney disease	121,483 (10.8)	2638 (13.7)	8580 (13.3)	34,066 (11.0)	76,199 (10.4)	<.001	.10
Depressive disorder	74,303 (6.6)	1432 (7.42)	4756 (7.4)	20,816 (6.7)	47,299 (6.5)	<.001	.04
Anxiety disorder	151,897 (13.5)	3047 (15.8)	9597 (14.8)	41,733 (13.4)	97,520 (13.4)	<.001	.07
Prediabetes	304,749 (27.1)	5148 (26.7)	17,635 (27.3)	82,890 (26.7)	199,076 (27.3)	<.001	.01
Body mass index (kg/m^2)	24.0 ± 3.1	24.0 ± 3.3	23.9 ± 3.3	23.9 ± 3.1	24.1 ± 3.0	<.001	.08
Waist circumference (cm)	79.5 ± 8.1	80.1 ± 8.6	79.6 ± 8.5	79.0 ± 8.2	79.6 ± 8.0	<.001	.13
Fasting glucose (mg/dL)	93.8 ± 11.1	93.5 ± 11.3	93.8 ± 11.3	93.6 ± 11.0	93.9 ± 11.1	<.001	.03
Systolic blood pressure (mmHg)	125.0 ± 16.0	125.8 ± 16.7	125.4 ± 16.7	124.3 ± 16.1	125.3 ± 15.9	<.001	.10
Diastolic blood pressure (mmHg)	76.8 ± 10.1	77.2 ± 10.4	76.9 ± 10.4	76.5 ± 10.2	76.9 ± 10.1	<.001	.07
Total cholesterol (mg/dL)	208.7 ± 37.9	206.8 ± 38.6	206.3 ± 38.3	207.7 ± 37.9	209.3 ± 37.9	<.001	.08
**Reproductive factors**							
Age at menarche (years)	16.4 ± 1.9	16.9 ± 2.1	16.6 ± 2.0	16.4 ± 1.9	16.4 ± 1.8	<.001	.23

© 2024 Ko B-J et al. *JAMA Network Open*.

Parity (yes)	1,105,857 (98.3)	18,784 (97.3)	63,205 (97.7)	304,574 (98.0)	719,294 (98.5)	<.001	.08
Breastfeeding (yes)	1,050,768 (93.4)	17,735 (91.8)	59,443 (91.9)	286,634 (92.2)	686,956 (94.0)	<.001	.09
Oral contraceptive use (yes)	172,166 (15.3)	2643 (13.7)	9525 (14.7)	48,128 (15.5)	111,870 (15.3)	<.001	.05
Menopausal hormone therapy (yes)	187,194 (16.6)	3660 (19.0)	12,181 (18.8)	58,918 (19.0)	112,435 (15.4)	<.001	.10

Abbreviations: MASD, maximum absolute standardized mean difference.
Data are presented as mean ± standard deviation or number (percentage).

© 2024 Ko B-J et al. *JAMA Network Open*.

eTable 2. Hazard ratios (95% confidence intervals) of type 2 diabetes according to age at menopause (three categories)

	N	Event	Person-years	IR[a]	Model 1[b]	Model 2[c]	Model 3[d]
<40	19,311	2337	154,304	15.2	1.21 (1.16–1.26)	1.13 (1.09–1.18)	1.13 (1.08–1.18)
40–44	64,700	6912	519,820	13.3	1.06 (1.04–1.09)	1.03 (1.01–1.06)	1.03 (1.01–1.06)
≥45	1,041,367	104,615	8,368,549	12.5	1 (reference)	1 (reference)	1 (reference)
P for trend					<.001	<.001	<.001

Abbreviations: HR, hazard ratio; CI, confidence interval; IR, incidence rate.
HRs (95% CIs) were calculated using multivariable Cox proportional hazards regression analysis.
[a]Incidence per 1,000 person-years.
[b]Model 1 was not adjusted.
[c]Model 2 was adjusted for age, income, place of residence, smoking status, alcohol consumption, physical activity, body mass index categories, hypertension, dyslipidemia, chronic kidney disease, depressive disorder, and anxiety disorder.
[d]Model 3 was adjusted for age, income, place of residence, smoking status, alcohol consumption, physical activity, body mass index categories, hypertension, dyslipidemia, chronic kidney disease, depressive disorder, anxiety disorder, age at menarche, parity, breastfeeding, oral contraceptive use, and menopausal hormone therapy.

© 2024 Ko B-J et al. *JAMA Network Open*.
