# Supplementary material for: Age at Menopause and Development of Type 2 Diabetes in Korea
Source: JAMA Netw Open. 2025 Jan 21;8(1):e2455388. doi: 10.1001/jamanetworkopen.2024.55388 (PMC11751743; doi:10.1001/jamanetworkopen.2024.55388)
Supplement: Supplement 2. — Data Sharing Statement [file jamanetwopen-e2455388-s002.pdf]

Data Sharing Statement

Ko. Age at Menopause and Development of Type 2 Diabetes in Korea. *JAMA Netw Open*. Published online January 21, 2025. doi:10.1001/jamanetworkopen.2024.55388

Data
Data available: No

Additional Information
Explanation for why data not available: The data that support the findings of this study are available from the Korean National Health Insurance Service but restrictions apply to the availability of these data, which were used under license for the current study, and so are not publicly available. Data are however available at https://nhiss.nhis.or.kr/bd/ay/bdaya001iv.do with the permission of Korean National Health Insurance Service.
